# Nutraceuticals or Food Supplements? Knowledge, Attitudes, and Behaviors Among Adults in Southern Italy

**DOI:** 10.3390/medsci14060609

**Published:** 2026-09-27

**Authors:** Giorgia Della Polla, Maria Catone, Vincenzo Monda, Francesco Napolitano

**Affiliations:** 1 Department of Experimental Medicine, University of Campania “Luigi Vanvitelli”, 80138 Naples, Italy; giorgia.dellapolla@unicampania.it (G.D.P.); maria.catone@unicampania.it (M.C.); 2 Department of Economics, Law, Cybersecurity, and Sports Sciences, University of Naples “Parthenope”, 80131 Naples, Italy; vincenzo.monda@uniparthenope.it

**Keywords:** behaviors, Italy, knowledge, nutraceuticals, survey

## Abstract

**Background/Objectives**: Nutraceuticals are increasingly consumed globally, yet public knowledge, attitudes, and behaviors toward these products remain inadequately characterized, particularly in Italy. This study aimed to assess the knowledge, attitudes, and behaviors regarding nutraceuticals among adults residing in the metropolitan area of Naples, Southern Italy, and to identify the factors associated with nutraceutical use and digital information-seeking behavior. **Methods**: A cross-sectional study was conducted between January and February 2026 using a self-administered questionnaire. **Results**: A total of 495 adults were enrolled. Nutraceutical use in the previous 12 months was reported by 37.8%, while the Internet or social networks were the most cited information sources (49.7%). Multivariate logistic regression showed that nutraceutical use was significantly associated with higher educational level, interest in participating in educational/informative initiatives on nutraceuticals, and non-use of the Internet or social networks to acquire information about nutraceuticals. Internet or social networks use to acquire information about nutraceuticals was associated with younger age, belief in nutraceutical necessity even in the case of a healthy diet, and disagreement with the idea that nutraceuticals are inherently safe because of natural origin. **Conclusions**: Misconceptions and limited risk awareness regarding nutraceuticals are widespread, indicating the need for clearer, evidence-based public communication. Interventions may combine routine counseling by healthcare professionals with initiatives to strengthen eHealth literacy, particularly for younger adults who frequently rely on digital resources. This combined approach may support safer and more informed decisions about nutraceutical use.

## 1. Introduction

In recent years, there has been a growing global interest in nutraceuticals. This term was coined in 1989 from nutrition and pharmaceutical to designate foods or food components providing health benefits beyond basic nutrition [1]. These compounds, including bioactive molecules, plant extracts, vitamins, minerals, and fatty acids, are increasingly used both for the prevention of chronic diseases and for the promotion of overall well-being [2]. The term functions as an umbrella label encompassing a heterogeneous range of preparations that are marketed and regulated under different names across jurisdiction, such as food supplements in the European Union, dietary supplements in the United States, and health functional foods in several Asian countries [3]. Therefore, the same product may fall under different regulatory requirements depending on where it is sold, and it complicates the comparison of prevalence estimates across studies [4]. Accordingly, nutraceuticals have no autonomous legal status in the European Union, and products falling within this description are placed on the market and regulated as food supplements under Directive 2002/46/EC, transposed in Italy by Legislative Decree 169/2004. This framework requires notification rather than pre-market authorization and does not demand evidence of clinical efficacy comparable to that required for medicinal products, while health claims are separately governed by Regulation (EC) No 1924/2006. The boundary between food and medicine consequently remains blurred in the perception of consumers, who may attribute therapeutic properties to products assessed only for safety as foods. Medicinal product authorization (Directive 2001/83/EC) requires establishing a dose-effect relationship and demonstrating clinical efficacy for a defined indication, whereas food supplement regulation (Directive 2002/46/EC) requires only that the finished product be safe, typically against a toxicologically derived upper level, with no requirement to demonstrate efficacy at the marketed dose. As a result, the same bioactive substance can appear in near-identical pharmaceutical-like forms while being subject to two entirely different evidentiary standards, with no visible cue allowing consumers to distinguish a clinically validated dose from one whose only demonstrated property is safety.

This regulatory ambiguity coexists with sustained expansion of the nutraceutical market, which is largely driven by the perception that such products are natural, effective and safe alternatives to conventional pharmaceuticals. Indeed, younger consumers who are more aware of nutraceutical concepts are more likely to seek such products in the market [5]. However, the widespread and sometimes unregulated use raises concerns such as self-medication, lack of professional supervision, and varying levels of scientific knowledge among consumers [6]. In fact, these products are not devoid of risk, as adverse events, hepatotoxicity associated with specific botanical preparations, and clinically relevant interactions with prescribed medications have been documented [7].

Additionally, consumers frequently do not disclose the use of these products to their physician or pharmacist, so that such interactions often remain undetected in routine clinical practice [8]. Evidence indicates that more than half of adults use dietary supplements and that a large majority consider them safe despite limited detailed awareness [6], suggesting that familiarity with these products does not translate into awareness of their risks.

For this reason, a growing body of international research has assessed knowledge, attitudes, and behaviors towards these products in different settings. Cross-sectional studies conducted in Bahrain [5], Poland [9], Iran [10], Spain [11], and Greece [12] have consistently reported a high prevalence of use alongside substantial knowledge gaps, particularly regarding adverse effects and interactions with medications.

However, a further dimension has emerged only recently. Health information is increasingly retrieved from digital sources. For example, social media platforms are widely used to promote these products, and commercial content, influencer endorsement, and peer testimonials circulate there alongside evidence-based information [13]. This has brought attention on eHealth literacy (eHL), that is the ability to critically appraise online health information and to assess its quality, reliability, and relevance to personal health decisions [14]. Nevertheless, the characteristics associated with digital information-seeking about these products in the adult population remain largely unexplored.

In Italy, the available evidence is limited. National data on food supplement use derive from surveys conducted more than a decade ago [15,16], and thus predate the current digital information environment. More recently, Calella et al. [17] investigated knowledge, attitudes, and practices concerning food supplements and nutraceuticals in Italian adults. However, their research question was focused on exercise practice and setting, and recruitment centered on individuals engaged in structured physical activity, resulting in a substantially younger sample. Moreover, individuals taking medications that could interfere with supplement use were excluded by design. The extent to which those findings apply to older adults who are not physically active in organized settings therefore remains unclear. Understanding the knowledge, attitudes, and behaviors of the general population toward nutraceuticals is crucial for public health. Assessing these dimensions provides insights into health literacy levels, perceived safety and efficacy, and trust in healthcare professionals. Such data are fundamental to guide targeted health education and communication strategies aiming to promote the rational and evidence-based use of nutraceuticals [10,18].

To the best of our knowledge, no Italian study has characterized knowledge, attitudes, and behaviors towards nutraceuticals in a general adult sample and examined the factors associated with the use of digital sources to obtain information about them. This double gap is relevant, because the two dimensions are plausibly interrelated. The channel through which individuals acquire information may be connected both to their beliefs about these products and to their consumption patterns, and it may define an entry point for any public health intervention.

The present study was therefore conducted to assess the knowledge, attitudes, and behaviors regarding nutraceuticals among adults residing in the metropolitan area of Naples, Southern Italy, and to identify the factors associated with nutraceutical use and the use of the Internet or social networks as a source of information about these products, with the goal of identifying potential gaps and opportunities to enhance public awareness and promote responsible and evidence-based use.

## 2. Materials and Methods

### 2.1. Study Population and Sampling

The survey was conducted between January and February 2026 in the metropolitan area of Naples, Italy. This area includes more than three million inhabitants with heterogeneous socio-demographic characteristics. A two-stage cluster sampling method was used to select a community-based sample of adults. In the first stage, from the official list of public preschools, primary, lower secondary, and high schools located in the metropolitan area, 5 schools were randomly selected. In the second stage, a total amount of 26 classes was selected using simple random sampling, and all parents aged ≥18 years or legal guardians of students enrolled in the selected classes were invited to participate. The school-based recruitment approach allowed access to a broad and socio-demographically diverse adult population. The minimum required sample size was calculated assuming a prevalence of 20% of individuals who use nutraceuticals [9,11,12,16,19,20], a 95% confidence level, and a 5% margin of error, yielding 246 participants. Prevalence estimates specifically for “nutraceuticals” as an autonomous construct are not available in the literature. As these products do not hold a distinct regulatory status from food/dietary supplements, the 20% estimate was derived from prevalence studies on food/dietary supplement consumption, under the assumption that this prevalence would represent a conservative and reasonably comparable basis for sample size calculation. To account for the two-stage cluster design, this figure was multiplied by a design effect of 2, resulting in a minimum requirement of 492 participants. Assuming an expected response rate of 50%, 991 questionnaires were distributed and 495 valid questionnaires were returned (49.9%), exceeding the minimum required sample size.

### 2.2. Data Collection

Before initiating data collection, the research team contacted the school principals through an official communication explaining the study objectives, methodology, and procedures and requesting authorization to conduct the survey. After approval was granted, teachers distributed sealed envelopes to students to deliver to their parents or guardians. Each envelope contained the following: an information sheet describing the purpose of the study and contact details; an anonymous self-administered questionnaire (Supplementary file: Questionnaire) to be filled out by only one parent; an informed consent form; two pre-addressed envelopes for the separate return of the completed questionnaire and the signed consent form. Participants were informed that the survey was voluntary and anonymous, that no personal identifiers would be collected, and that all data would be handled confidentially. No financial or other incentives were provided. Completed materials were returned either to teachers or directly to collection boxes placed in the schools. The study protocol was approved by the Ethics Committee Campania 2 (protocol number 0028598/i) on 27 October 2025.

### 2.3. Survey Instrument

The questionnaire was specifically developed for this study based on previous surveys regarding nutraceutical and dietary/food supplement use [9,21,22]. Cronbach’s alpha was calculated on the attitude items, yielding a value of 0.69.

The first section collected socio-demographic, anthropometric, and clinical, information, including year of birth, gender, marital status, parental status, educational level, occupation, height, weight, presence of chronic diseases, and medication use.

The second section assessed participants’ knowledge about nutraceuticals. At the beginning of the section, participants were given the following standardized definition: “a nutraceutical is a food or part of a food that provides medical or health benefits, including the prevention and/or treatment of a disease”. This broad formulation was chosen to reproduce the framing of “nutraceuticals” commonly encountered by consumers in commercial and lay discourse, rather than the stricter definition of permissible health claims under Regulation (EC) No 1924/2006, which prohibits claims referring to the treatment or cure of disease for food-marketed products. This choice allowed us to assess knowledge, attitudes, and behaviors under conditions resembling real-world exposure, including participants’ tendency to accept an implied therapeutic function uncritically. Throughout the manuscript, however, the term nutraceutical is used in the narrower operational sense of food-derived products containing concentrated bioactive constituents (vitamins and minerals, omega-3 fatty acids, probiotics, botanical and phytotherapeutic extracts, and compounds such as curcumin, phytosterols, and melatonin) taken in pharmaceutical-like dosage forms for health promotion or disease prevention, and placed on the Italian market within the regulatory category of food supplements. Since nutraceuticals and food supplements are not legally differentiated in Italy, no separate explanation distinguishing the two categories was provided. Additionally, food supplement is used when referring to the EU regulatory category (Directive 2002/46/EC) and dietary supplement when reporting findings from studies conducted under the US regulatory framework to preserve the terminology of the original sources. Participants were asked whether they had ever heard of nutraceuticals (yes/no) and, in case of affirmative response, to indicate the person or source of information whom they had first become aware of nutraceuticals. Subsequently, they were asked to identify nutraceuticals from a list of six products (omega-3 fatty acids, probiotics, curcumin, phytosterols, melatonin, and vitamins/minerals), with the option to select multiple options or to answer “I don’t know”. Participants were also asked to select, from a predefined list of seven options, the health benefits they recognized as being associated with nutraceuticals (disease prevention, general wellbeing improvement, memory enhancement, sleep support, vision improvement, immune system strengthening, and metabolism acceleration), with the possibility of selecting multiple options or to answer “I don’t know”. Additional items explored whether nutraceuticals could replace a balanced diet (yes/no/don’t know), whether their use may lead to adverse effects (yes/no), with respondents asked to specify in the affirmative case, and whether they could interact with medications (yes/no/don’t know). Finally, participants were asked to identify, from a list of predefined categories, which population group(s) should exercise particular caution when using nutraceuticals due to the risk of adverse effects (pregnant women, older adults, children, and individuals with chronic diseases), or to select “nobody” or “I don’t know”.

The third section assessed attitudes toward nutraceuticals. Participants first rated their perceived usefulness and safety of nutraceuticals on two 10-point Likert scales (1 = “not at all”/10 = “very much”). Subsequently, eight statements assessed the belief in the role of nutraceuticals in preventing chronic diseases, perceived necessity of nutraceuticals even in the case of a healthy diet, importance of medical consultation prior to nutraceutical use, perceived risk associated with unsupervised nutraceutical use, belief that the natural origin of nutraceuticals renders them inherently safe, perception of nutraceuticals as cost-prohibitive, perceived public awareness of nutraceutical benefits and risks, and interest in participating in educational or informational initiatives on nutraceuticals. Agreement with each statement was rated on a 5-point Likert scale (1 = “strongly disagree”/5 = “strongly agree”).

The fourth section investigated behaviors related to nutraceutical use. Participants were asked whether they had used over-the-counter (OTC) medications in the previous 12 months (yes/no) and, if so, the reason(s) for their use (pain, fever, cold/flu symptoms, or allergies). Subsequently, participants were asked whether they had used nutraceuticals in the previous 12 months (yes/no); those who responded affirmatively were further asked about what kind of nutraceuticals they used, frequency of intake (daily, weekly, monthly, or less than once a month), reason(s) for use (disease prevention, treatment of mild symptoms, immune system strengthening, aesthetic purposes, or dietary supplementation), source(s) of recommendation (physician, pharmacist, the Internet, family/friends, or nobody), and place(s) of purchase (pharmacy, para-pharmacy, herbalist shops, supermarket, or online). Finally, participants were asked whether they had ever experienced adverse effects from nutraceuticals (yes/no/don’t know), and those who responded affirmatively were asked to specify the nature of the adverse effects.

The final section explored sources of information (physician, pharmacist, Internet, social networks, TV/newspaper/radio, family/friends) and perceived need for further information on nutraceuticals with a “yes/no” answer.

The questionnaire was pilot-tested on a sample of 20 adults with sociodemographic characteristics comparable to those of the target population to assess clarity, comprehensibility, and completion time. No changes were required following the pilot. These individuals were not included in the final study sample.

### 2.4. Statistical Analysis

Data were analyzed using Stata version 19 (StataCorp LLC, College Station, TX, USA) [23]. Descriptive statistics were used to summarize participants’ characteristics. Categorical variables were presented as frequencies and percentages, and continuous variables as means and standard deviations. Denominators differ across items because the questionnaire used filtered questions, so that each item refers to a specific subsample. The reference population is therefore reported as n/N for each estimate.

Bivariate analyses (chi-square test for categorical variables and Student’s *t*-test for continuous variables) were performed to assess associations between independent variables and each outcome of interest. All variables with a *p*-value ≤ 0.25 in bivariate analyses were included in two multivariable stepwise logistic regression models according to the Hosmer and Lemeshow’s model building strategy [24]. Two different outcomes were analyzed: Use of nutraceuticals in the last 12 months (no = 0; yes = 1) (Model 1); Use of the Internet or social networks to acquire information about nutraceuticals (no = 0; yes = 1) (Model 2). For Model 1, the following tested independent variables were included in the final model: educational level (0 = high school degree or less/1 = baccalaureate/graduate degree), use of medication (0 = no/1 = yes), use of the Internet or social networks to acquire information about nutraceuticals (0 = no/1 = yes), and interest in participating in educational/informative initiatives on nutraceuticals (continuous). For Model 2, the following tested independent variables were included in the final model: age (continuous), BMI (continuous), the belief that nutraceuticals are necessary even in case of healthy diet (continuous), the belief that nutraceuticals are inherently safe due to their natural origin (continuous), and the need to obtain more information about nutraceuticals (0 = no/1 = yes). Odds ratios (ORs) with 95% confidence intervals (CIs) were calculated. All statistical tests were two-tailed, and *p*-values ≤ 0.05 were considered statistically significant. Cases with missing data for the variables included in the analyses were excluded using listwise deletion.

## 3. Results

Among the 991 questionnaires distributed, 495 were returned and considered valid for analysis, yielding a response rate of 49.9%. The mean age of the sample was 46.8 ± 6.8 years. Most participants were female (71.8%), a large majority were married or cohabiting with a partner (87.4%), and almost the entire sample (97.7%) reported having at least one child with a mean of 2.1 ± 0.9. Overall, 77.5% had a high school diploma or less, and 64% were employed. The mean BMI was 26.1 ± 4.3. Approximately one in five of participants had at least one chronic medical condition (22.9%) and 25.7% of the participants reported regularly taking medications. Table 1 summarizes the participants’ main sociodemographic, anthropometric, and anamnestic characteristics.

### 3.1. Knowledge About Nutraceuticals

Knowledge of nutraceuticals appeared generally limited among participants. Overall, only 32% (154/481) of participants had ever heard of the term “nutraceutical”, with the remaining 68% (327/481) reporting no prior awareness. Among those who had heard of the term (n = 154), 33.1% (51/154) did not report the person or source from whom they first heard about them. When reported, physicians or nutritionists were the most frequently cited (31.2%; 48/154), followed by the Internet or social networks (13%; 20/154), television or other mass media (9.7%; 15/154), and family and friends (7.8%; 12/154). The remaining sources were the respondent’s own studies (3.9%; 6/154), colleagues (3.2%; 5/154), and magazines or books (2.6%; 4/154). When identifying nutraceuticals (354/482), the most frequently selected were omega-3 fatty acids (68.1%; 241/354) and vitamins/minerals (66.1%; 234/354), followed by probiotics (44.1%; 156/354), curcumin (21.8%; 77/354), melatonin (18.9%; 67/354), and phytosterols (7.1%; 25/354). However, 26.6% (128/482) were unable to identify any nutraceutical product.

Regarding perceived health benefits (n = 372), the most commonly selected were general wellbeing improvement (72.3%; 269/372), immune system strengthening (55.4%; 206/372), and disease prevention (45.2%; 168/372), followed by memory enhancement (24.2%; 90/372), sleep support (19.6%; 73/372), metabolism acceleration (15.3%; 57/372), and vision improvement (11.3%; 42/372).

Knowledge of potential risks was particularly limited. When asked whether nutraceuticals could cause adverse effects, 70.8% (298/421) responded “no”, while 29.2% (123/421) acknowledged this possibility. As this item did not include a “don’t know” option, the proportion answering “no” may encompass both respondents who actively excluded the occurrence of adverse effects and respondents who were uncertain. Among the possible adverse effects, participants mainly reported gastro-intestinal effects and allergies. Similarly, awareness of potential drug interactions was low, as 57.3% (264/461) answered “I don’t know”, 14.3% (66/461) incorrectly responded “no”, and 28.4% (131/461) correctly recognized that nutraceuticals may interact with medications. Regarding the misconception that nutraceuticals can replace a balanced diet, 54.1% (260/481) correctly answered “no”, while 38.5% (185/481) responded “I don’t know” and 7.5% (36/481) incorrectly believed they could substitute for proper nutrition. When asked to identify population groups requiring caution when consuming nutraceuticals due to possible adverse effects, 56.1% (268/478) selected at least one group, whereas 37.4% (179/478) selected “I don’t know” and 6.5% (31/478) indicated that no population group is at risk. The most frequently recognized at-risk groups were pregnant women (64.2%; 172/268) and individuals with chronic diseases (63.4%; 170/268), followed by older adults (48.9%; 131/268), and children (43.7%; 117/268).

### 3.2. Attitudes About Nutraceuticals

On a 10-point scale, participants rated the perceived usefulness of nutraceuticals with a mean score of 6.6 ± 2.1 (range 1–10), while perceived safety received a slightly lower mean score of 6.5 ± 2.1 (range 1–10). On a 5-point Likert scale (1 = strongly disagree/5 = strongly agree), participants reported a moderate level of agreement regarding nutraceuticals’ potential to prevent chronic diseases (mean 3.2 ± 0.9), with 4.4% strongly agreeing. Agreement was slightly higher for the statement on the necessity of nutraceuticals even in the presence of a healthy diet (mean 3.4 ± 0.9), with 4.8% strongly agreeing. Perceived importance of medical consultation showed the highest mean score (mean 3.7 ± 1.0), and 18.8% of participants strongly agreed that consulting a physician before taking nutraceuticals is important. Similarly, agreement that unsupervised nutraceutical use is associated with health risks was moderate (mean 3.3 ± 1.0), with 12.1% strongly agreeing.

Lower agreement emerged for the belief that the natural origin of nutraceuticals renders them inherently safe (mean 2.9 ± 0.9), although 2.8% strongly agreed. Cost was perceived as a barrier to some extent (mean 3.1 ± 0.9), with 5.5% strongly agreeing that nutraceutical prices are high and that they are unwilling to pay the additional expense. Perceived public awareness was believed to be low (mean 2.8 ± 0.9), with only 3.5% strongly agreeing that the general population is aware of both benefits and risks. Finally, willingness to participate in educational/informative initiatives on nutraceuticals was moderate (mean 3.2 ± 1.0), with 7.6% strongly agreeing.

### 3.3. Behaviors About OTC Medication and Nutraceuticals

In the last 12 months, 80.4% (389/484) of participants reported OTC medication use; among those who specified the reason for OTC medications use (n = 385), the most common was pain (72.2%; 278/385), followed by flu/cold symptoms (42.1%; 162/385), fever (25.2%; 97/385) and allergies (14.8%; 57/385). Regarding nutraceuticals use, 37.8% (182/482) reported having used them in the last 12 months, while 62.2% (300/482) had not. Among nutraceutical users, more than two thirds of participants did not specify the type of product used (70.3%; 128/182), reporting it only as generic food supplements. Among those who specified at least one product (n = 54), the most common were vitamins (48.1%; 26/54), probiotics (24.1%; 13/54), phytotherapeutic agents or botanical products (18.5%; 10/54), minerals like magnesium or potassium (16.7%; 9/54), omega-3 fatty acids (11.1%; 6/54), and melatonin (3.7%; 2/54).

Frequency of consumption varied considerably among users who answered this item (n = 178). Nearly half (44.9%; 80/178) used nutraceuticals less than once a month, 19.7% (35/178) monthly, 6.7% (12/178) weekly, and 28.7% (51/178) daily. The main motivations for nutraceutical use (n = 181) were disease prevention (36.5%; 66/181), strengthening immune system (35.9%; 65/181), and dietary supplementation (24.9%; 45/181), followed by treatment of mild symptoms (23.2%; 42/181) and cosmetic purposes (3.3%; 6/181). Among users who answered the item on sources of recommendation (n = 179), 16.8% (30/179) reported having received no recommendation from any source. Among the 149 users who indicated at least one source, and for whom more than one answer was possible, physicians (63.1%; 94/149) and pharmacists (32.9%; 49/149) were the most frequently cited, while the Internet (11.4%; 17/149) and family or friends (7.4%; 11/149) were less commonly reported. Pharmacies were by far the predominant point of purchase (73.2%; 131/179), followed by online sources (16.2%; 29/179), para-pharmacies (14%; 25/179), herbalist shops (3.9%; 7/179) and supermarkets (7.8%; 14/179). Among users who answered the item on adverse effects (n = 180), 71.1% (128/180) reported never having experienced any, 21.7% (39/180) were uncertain, and 7.2% (13/180) reported having experienced them, including nausea, diarrhea, and tachycardia.

A multivariable logistic regression model was fitted to assess which variables were more likely to affect participants’ use of nutraceuticals (Table 2): participants interested in participating in educational/informative initiatives on nutraceuticals (OR = 1.56; 95% CI = 1.23–1.98), who did not use the Internet or social networks to acquire information about nutraceuticals (OR = 0.48; 95% CI = 0.29–0.78), and who had a baccalaureate/graduate degree (OR = 2.12; 95% CI = 1.19–3.78) were more likely to have used nutraceuticals in the last 12 months.

### 3.4. Sources of Information About Nutraceuticals

Information sources regarding nutraceuticals varied widely among participants. Overall, 69% (312/452) had acquired information about these products from at least one source, while 31% (140/452) reported no information sources used. Among those who had used at least one information source (n = 312), the Internet or social networks were the most commonly reported (49.7%; 155/312), followed by physicians (30.8%; 96/312) and pharmacists (19.9%; 62/312). Family/friends (18.9%; 59/312) were also mentioned. Traditional media (television/newspapers/radio) were cited by 14% (44/312) of participants. Finally, slightly more than half of the sample (53.4%; 227/425) expressed the need for more information on nutraceuticals. A multivariable logistic regression model was fitted to assess which variables were more likely to affect participants’ use of the Internet or social networks to acquire information about nutraceuticals (Table 2): younger participants (OR = 0.93; 95% CI = 0.9–0.97), who agreed that nutraceuticals are necessary even in case of a healthy diet (OR = 1.52; 95% CI = 1.12–2.07), and who did not agree with the belief that nutraceuticals are inherently safe because of their natural origin (OR = 0.69; 95% CI = 0.51–0.92) were more likely to use the Internet or social networks to acquire information about nutraceuticals.

## 4. Discussion

This study explored knowledge, attitudes, and behaviors regarding nutraceutical use in an Italian adult sample and identified factors associated with its consumption and digital information-seeking behaviors. Specifically, nutraceutical use in the last 12 months was reported by 37.8% of participants, and a large proportion accessed information on nutraceuticals via the Internet or social networks (49.7%).

Reported prevalences of nutraceuticals use in adult populations vary widely across countries: frequent use has been documented among Greek adults, with variability depending on sociodemographic and lifestyle characteristics [25], whereas a Polish survey reported that 10% of the population consumed food supplements [9], and high rates have been consistently observed among US adults [26]. These figures, however, are not directly comparable with our findings. It should be noted that studies differ in the product categories considered, which range from nutraceuticals to food or dietary supplements as defined under different regulatory frameworks, as well as in the populations examined and in the study designs adopted [27]. Our estimate should therefore be read as referring specifically to self-reported use over the previous 12 months, among adults recruited through schools in a single metropolitan area, of products falling within the operational definition adopted in this study, rather than as a value directly comparable to national prevalences reported elsewhere. However, a noteworthy finding is the apparent mismatch between awareness and use. Only a minority had previously heard about nutraceuticals, yet more than one-third reported use in the last 12 months. This suggests that many individuals may consume products commonly framed as “supplements” without recognizing them as nutraceuticals, complicating the interpretation of self-reported knowledge. Additionally, “nutraceutical” is not a harmonized legal category, whereas these products are marketed and regulated as food supplements under European Union rules [28]. This context may contribute to limited recognition of the term “nutraceutical” despite widespread exposure to supplement products [28]. Beyond terminology, the findings evidenced critical knowledge gaps concerning risk-related domains, as most respondents did not acknowledge the possibility of adverse effects and were uncertain about drug-nutraceuticals interactions. These findings represent a significant public health concern, as unsupervised nutraceutical use in the absence of risk awareness may expose vulnerable individuals to preventable harm. Nutraceuticals contain constituents that are capable of producing adverse effects in susceptible individuals, including clinically relevant pharmacokinetic interactions with concurrently administered medications [7,29]. Notably, risks have been documented for the categories most frequently recognized by our participants: for example, omega-3 supplements may potentiate anticoagulant therapy and promote bleeding, and excessive intake of fat-soluble vitamins and minerals has been associated with toxicity ranging from neuropathy to adverse effects on bone health [7]. Because consumers rarely disclose nutraceuticals use to their physicians, the potential for unrecognized interactions is substantial. In Italy, adverse reactions to food supplements and herbal products are monitored by the Phytovigilance System coordinated by the Istituto Superiore di Sanità, which by October 2020 had collected 2315 spontaneous reports, approximately one third concerning serious reactions involving hospitalization, life-threatening events or death, with concomitant medication use indicated in almost 40% of cases [30]. Since these products are largely self-selected, and 16.8% of users in our sample reported no recommendation from any source, gaps in risk awareness translate directly into unsupervised exposure. In fact, these findings are consistent with a broader pattern documented across multiple countries, whereby consumer familiarity with products does not translate into awareness of their risks [5]. It is noteworthy that the belief that nutraceuticals are inherently safe because of natural origin is a well-documented misconception that healthcare providers must actively address during patient counseling [31]. Additionally, the regulatory asymmetry provides a possible explanation for the apparent contradiction observed in our sample between a relatively high perceived usefulness and limited risk awareness. In the absence of a distinctive regulatory signal separating products with demonstrated efficacy from products whose only supporting evidence concerns safety, consumers lack the tools to correctly calibrate their therapeutic expectations.

The regression model identified three covariates significantly associated with reporting nutraceutical use in the last 12 months: higher educational attainment (baccalaureate/graduate degree), non-use of the Internet or social networks as a source of information on nutraceuticals, and interest in participating in educational/informative initiatives.

Educational level has repeatedly emerged as a predictor of preventive health behaviors, including dietary/food supplement and nutraceutical use [11,32,33]. Individuals with higher education may demonstrate greater engagement with health information and preventive strategies, even if knowledge gaps persist [25]. Contrary to the assumption that online exposure drives nutraceutical consumption, our model shows that users were less likely to rely on the Internet or social network as their information source. This may reflect the role of healthcare professionals in guiding use, as our findings show that physicians and pharmacists were the most frequently cited sources of recommendation among nutraceutical users, consistent with evidence showing that physician recommendation increases the likelihood of supplement use [34,35]. That nutraceutical users expressed greater interest in educational events may indicate a proactive prevention orientation. Studies consistently show that individuals who are more engaged in their health are also more likely to seek additional information and interventions [36,37].

The second model revealed that younger age, agreement that nutraceuticals are necessary even in the case of a healthy diet and disagreement with the belief that the natural origin of nutraceuticals renders them inherently safe are associated with the Internet or social network use as sources of information. The Internet, nowadays, represents the primary information source across age groups [38,39]. Contextually, this habit stands out as an emerging public health issue, as online information is not always reliable and requires users to have critical skills to navigate in digital environments when seeking health information [38,39]. In our study, online information-seeking was associated not with generic trust in nutraceuticals but with a combination of health motivation (the belief that nutraceuticals are necessary even alongside a healthy diet) and skepticism (disagreement with the assumption that natural origin implies safety). One possible explanation is that digital environments expose users to a high volume of competing narratives, including commercial promotion, influencer content, peer testimonials, and evidence-based guidance [13], although our data do not allow this mechanism to be tested. These considerations point to eHL as a potentially relevant framework for interpreting our findings [14]. We did not assess eHL in this study, and its role therefore remains hypothetical. Nonetheless, the observation that younger adults were more likely to seek nutraceutical information online is of interest given the documented circulation of dietary supplement misinformation on social media platforms [39,40]. Additionally, younger individuals have repeatedly been found to use the Internet and social network as primary health information sources [8,22]. Practically, healthcare providers should be equipped to address patients’ digitally acquired beliefs about nutraceuticals, acknowledging the complexity of online information environments. Aligning counseling strategies with the digital information landscape may be an effective approach to promoting evidence-based nutraceutical use in the general population [41,42].

Some limitations should be acknowledged when interpreting the findings. The school-based sampling strategy may have introduced a selection bias since the sample was composed of parents or legal guardians of school-aged children and/or adolescents. This approach systematically excludes adults without school-enrolled children, including childless adults, individuals whose children have already completed schooling, and elderly individuals, and it likely explains the near-universal presence of parental status (97.7%) and the marked female predominance (71.8%) observed in our sample, as questionnaires were more frequently completed by mothers. The sample was limited to the metropolitan area of Naples, which may restrict generalizability to other Italian populations and particularly to rural or less urbanized contexts. The cross-sectional design precludes causal inference, and the self-reported nature of the data is susceptible to social desirability bias and recall bias. The definition administered to participants was broader than the operational definition adopted in the analyses, as it referred generically to foods or food components providing health benefits, and could in principle encompass fortified foods. The closed-response items, however, listed only concentrated preparations, and the finding that most users described their products generically as dietary supplements suggests that responses largely refer to the category of food supplements. The results should be interpreted accordingly. Additionally, the item assessing the perceived occurrence of adverse effects was framed as a dichotomous yes/no question and did not include a “don’t know” option. Respondents who were uncertain may have been constrained to answer “no”, and the proportion answering negatively should not be interpreted as active denial of risk. However, the two items that did include a “don’t know” option yielded convergent findings, with only 28.4% correctly recognizing potential drug interactions and 56.1% identifying at least one at-risk population group, supporting the overall conclusion of limited risk awareness. Finally, the BMI was derived from self-reported height and weight, a method known to systematically underestimate true BMI. This bias may have attenuated or distorted the estimated association between BMI and the Internet/social network use as an information source in Model 2, where this variable reached borderline significance. Despite these limitations, this study provides a timely and detailed characterization of nutraceutical knowledge, attitudes, and behaviors in an Italian urban adult population, with implications for both clinical practice and public health policy.

## 5. Conclusions

In conclusion, the findings of this study showed that nutraceutical use is common and significantly associated with higher educational attainment, preventive orientation, and not using the Internet or social networks as a source of information about these products. On the other hand, being younger, believing that nutraceuticals are necessary even in case of a healthy diet, and not believing that their natural origin renders them inherently safe were associated with digital information-seeking behavior about nutraceuticals. Public health strategies may consider strengthening the role of healthcare providers in nutraceutical counseling and promoting evidence-based communication across the adult population, while initiatives supporting eHL skills appear particularly relevant for the more digitally engaged individuals, among whom younger age emerged as an associated factor. However, it is also fundamental to address the underlying regulatory ambiguity that places nutraceuticals in a grey zone. At the policy level, greater coordination among national regulatory authorities represents a necessary complement to communication and educational strategies.

## Figures and Tables

**Table 1:** Socio-demographic, anthropometric, and anamnestic characteristics of the participants.

Characteristics	N	Mean (±SD)	%
Age, in years		46.8 ± 6.8	
Gender			
Male	136		28.2
Female	346		71.8
Marital status			
Married/cohabited with a partner	422		87.4
Unmarried/separated/divorced/widowed	61		12.6
Having at least one child		2.1 ± 0.9	
Yes	464		97.7
No	11		2.3
Educational level			
High school degree or less	376		77.5
Baccalaureate/graduate degree	109		22.5
Employment status			
Unemployed	158		36
Employed	281		64
BMI level		26.1 ± 4.3	
Chronic medical condition			
Yes	109		22.9
No	368		77.1
Medication use			
Yes	121		25.7
No	349		74.3

Number for each item may not add up to the total number of the study population due to missing values.

**Table 2:** Results of multivariate logistic regression analysis to investigate the factors associated with the outcomes of interest.

Variable		OR	SE	95% CI	*p*
	Model 1. Using of nutraceuticals in the last 12 months Log likelihood = −184.98, χ^2^ = 34.35 (4 df), *p* < 0.0001				
Interest in participating in educational/informative initiatives on nutraceuticals		1.56	0.19	1.23–1.98	<0.001
Not having used Internet or social networks to acquire information about nutraceuticals		0.48	0.12	0.29–0.78	0.003
Having a baccalaureate/graduate degree		2.12	0.62	1.19–3.78	0.011
Having used medication		1.58	0.44	0.91–2.74	0.103
	Model 2. Having used the Internet or social networks to acquire information about nutraceuticals Log likelihood = −160.74, χ^2^ = 28.78 (5 df), *p* < 0.0001				
Younger age		0.93	0.02	0.9–0.97	0.002
Believing that nutraceuticals are necessary even in case of healthy diet		1.52	0.24	1.12–2.07	0.008
Not believing that nutraceuticals are inherently safe because of natural origin		0.69	0.1	0.51–0.92	0.013
Having lower BMI		0.94	0.03	0.88–1.01	0.062
Needing more information about nutraceuticals		1.44	0.39	0.85–2.44	0.179

## Data Availability

The anonymous data presented in this study are available on request from the corresponding author. Access to these data is restricted in order to safeguard the privacy and confidentiality of the study participants, in accordance with the informed consent terms.

## Supplementary Materials

The following supporting information can be downloaded at: https://www.mdpi.com/article/10.3390/medsci14060609/s1. Supplementary file: Questionnaire.

## Abbreviations

The following abbreviations are used in this manuscript:

BMI	Body Mass Index
CIs	Confidence Intervals
df	Degrees of freedom
eHL	eHealth literacy
OTC	Over-the-counter
OR	Odds Ratios
SD	Standard Deviation
SE	Standard Error
