# Supplementary material for: Nutraceuticals or Food Supplements? Knowledge, Attitudes, and Behaviors Among Adults in Southern Italy
Source: Med Sci (Basel). 2026 Sep 27;14(6):609. doi: 10.3390/medsci14060609 (PMC13706832; doi:10.3390/medsci14060609)
Supplement: Supplementary file 1 [file medsci-14-00609-s001.zip › medsci-4500428-supplementary.pdf]

KNOWLEDGE, ATTITUDES AND BEHAVIORS REGARDING NUTRACEUTICALS IN THE GENERAL POPULATION

The Department of Experimental Medicine of the University of Campania "Luigi Vanvitelli" has planned a survey to assess the population's knowledge, attitudes and behaviours regarding nutraceuticals. The questionnaire is anonymous. The information provided does not make it possible to trace the person who completed it. Pursuant to Legislative Decree 101/2018 on the protection of personal data, the data will be processed in aggregate form only and no personal details will be disclosed. By answering the questionnaire, you give your consent to participate and to the use and publication of the data.

MARK ONE ANSWER WITH AN X OR, WHERE INDICATED, GIVE A BRIEF ANSWER

A. SOCIO-DEMOGRAPHIC, MEDICAL HISTORY AND OCCUPATIONAL CHARACTERISTICS

The questions in this section aim to collect information on your socio-demographic, medical history and occupational characteristics.

A1. In what year were you born? ____

A2. What is your gender? ☐ Male ☐ Female ☐ Other ☐ Prefer not to answer

A3. What is your marital status? ☐ Married ☐ Cohabiting ☐ Single ☐ Divorced ☐ Widowed

A4. Do you have children? ☐ No ☐ Yes, how many? _______

A5. What is your highest educational qualification? ☐ Lower secondary school certificate ☐ Upper secondary school diploma ☐ Bachelor's degree ☐ Master's degree ☐ Other ____

A6. What is your current occupation? ______________________________

A7. What is your weight in kg? (give the last measured value) _______kg

A8. What is your height in cm? (give the last measured value)___________cm

A9. Do you suffer from any chronic disease? ☐ No ☐ Yes, please specify (more than one possible) __________

A10. Do you take any medication? ☐ No (go to B1) ☐ Yes, please indicate which and for how long

Medication	Duration of treatment in days/months/years

A. KNOWLEDGE

The following questions aim to assess your knowledge of nutraceuticals.

A nutraceutical is a food or part of a food that provides medical or health benefits, including the prevention and/or treatment of a disease

B1. Have you ever heard of nutraceuticals?

☐ No ☐ Yes, from whom?____________

B2. In your opinion, which of the following products is a nutraceutical? (more than one answer possible)

☐ Omega-3 ☐Probiotics ☐Curcumin ☐ Phytosterols ☐Melatonin ☐Vitamins/minerals ☐ Don't know ☐ Other _______

B3. In your opinion, what are the main positive effects of nutraceuticals on health? (more than one answer possible)

☐ Disease prevention ☐ Improvement of general well-being ☐ Improvement of memory ☐ Sleep support ☐ Improvement of eyesight ☐ Strengthening of the immune defences ☐ Speeding up of metabolism ☐ Don't know ☐ Other ________________________

B4 Can nutraceuticals replace a balanced diet? ☐ Yes ☐ No ☐ Don't know

B5. Can taking nutraceuticals lead to adverse effects? ☐ No ☐ Yes, which ones?________________________

B6. Can nutraceuticals interact with certain medications? ☐ Yes ☐ No ☐ Don't know

B7. In your opinion, which population groups should pay particular attention to taking nutraceuticals because of possible adverse effects? (more than one answer possible)

☐ Pregnant women ☐ Older adults ☐Children ☐ People with chronic conditions ☐No one ☐Don't know ☐ Other ____

B. ATTITUDES

The following questions aim to assess your attitudes towards nutraceuticals.

C1. On a scale from 1 to 10, how useful do you consider nutraceuticals to be for health?

Not at all 1 2 3 4 5 6 7 8 9 10 **Very much**

C2. On a scale from 1 to 10, how safe do you consider nutraceuticals to be?

Not at all 1 2 3 4 5 6 7 8 9 10 **Very much**

For the following statements, please indicate your level of agreement.

	Strongly disagree	Disagree	Uncertain	Agree	Strongly agree
**C3. I believe that nutraceuticals can help prevent chronic diseases (diabetes, cardiovascular disease, cancer, osteoporosis)**	☐	☐	☐	☐	☐
**C4. I believe that nutraceuticals are necessary even when following a healthy diet**	☐	☐	☐	☐	☐
**C5. It is important to consult a doctor before taking a nutraceutical**	☐	☐	☐	☐	☐
**C6. Using nutraceuticals without medical supervision is risky**	☐	☐	☐	☐	☐
**C7. I believe that nutraceuticals, being natural products, can be taken without concern**	☐	☐	☐	☐	☐
**C8. The price of nutraceuticals is high and I am not willing to bear an additional expense for these products**	☐	☐	☐	☐	☐
**C9. I believe that the general public is aware of the benefits and possible risks of nutraceuticals**	☐	☐	☐	☐	☐
**C10. I would like to take part in training/information activities on nutraceuticals**	☐	☐	☐	☐	☐

C. <u>BEHAVIORS</u>

The following questions aim to assess your behaviours regarding nutraceuticals.

D1. In the last 12 months, have you taken any over-the-counter medicines (such as painkillers or anti-inflammatories)? ☐ No (go to D3) ☐ Yes

D2. For what reason did you use over-the-counter medicines? (more than one answer possible) ☐ Pain (headache, muscle pain, menstrual pain) ☐ Fever ☐ Cold/flu symptoms ☐ Allergies ☐ Other ____

D3. In the last 12 months, have you used nutraceuticals? ☐ No (go to E1) ☐ Yes, please specify which______

D4. How often do you take nutraceuticals? ☐ Daily ☐ Weekly ☐ Monthly ☐ Less than once a month

D5. For what reason? (more than one answer possible) ☐ Prevention ☐ Treatment of mild symptoms ☐ Boosting the immune defences ☐ Cosmetic concerns ☐ Dietary supplementation ☐ Other _____

D6. Who recommended them? (more than one answer possible) ☐ Medical Doctor ☐ Pharmacist ☐Internet ☐ Friends/family
☐ No one ☐ Other ____________

D7. Where did you buy them? (more than one answer possible) ☐ Pharmacy ☐ Parapharmacy ☐ Herbalist's shop ☐ Supermarket ☐ Online ☐ Other ___

D8. Have you ever experienced adverse effects from nutraceuticals? ☐ No ☐ Don't know ☐ Yes, please specify____

D. <u>SOURCES OF INFORMATION</u>

The following questions aim to assess your sources of information on nutraceuticals.

E1. From which source(s) have you obtained information on nutraceuticals? (more than one answer possible)
☐ None ☐ Medical Doctor ☐ Pharmacist ☐Internet ☐ Social network ☐ TV/Newspaper/Radio ☐ Friends/family ☐ Other ____

E2. Do you believe to need additional information about nutraceuticals? ☐ No ☐ Yes

The questionnaire is now complete. If you would like to add a comment, please use the space below.

THANK YOU FOR TAKING PART IN THE SURVEY
